# Open or closed: Changes in ductus arteriosus flow patterns at birth using 4D flow MRI in newborn piglets

**DOI:** 10.14814/phy2.14999

**Published:** 2021-08-26

**Authors:** Eric M. Schrauben, Jack R. T. Darby, Mary J. Berry, Brahmdeep S. Saini, Megan Quinn, Stacey L. Holman, Emma L. Bradshaw, Mitchell C. Lock, Sunthara R. Perumal, Steven K. S. Cho, Tanroop Aujla, Mike Seed, Christopher K. Macgowan, Janna L. Morrison

**Affiliations:** ^1^ Translational Medicine Hospital for Sick Children Toronto ON Canada; ^2^ Early Origins of Adult Health Research Group, Health and Biomedical Innovation UniSA: Clinical and Health Sciences University of South Australia Adelaide SA Australia; ^3^ Centre for Translational Physiology & Department of Pediatrics and Child Health University of Otago Wellington New Zealand; ^4^ Heart Centre Hospital for Sick Children Institute of Medical Science Faculty of Medicine University of Toronto Toronto ON Canada; ^5^ Preclinical Imaging and Research Laboratories South Australian Health & Medical Research Institute Adelaide SA Australia; ^6^ Department of Physiology, Faculty of Medicine University of Toronto Toronto ON Canada; ^7^ Division of Cardiology Department of Paediatrics Hospital for Sick Children University of Toronto Toronto ON Canada; ^8^ Department of Biophysics University of Toronto Toronto ON Canada

**Keywords:** 4D flow MRI, ductus arteriosus, newborn

## Abstract

The ductus arteriosus (DA) functionally closes during the transition from fetal to postnatal life because of lung aeration and corresponding cardiovascular changes. The thorough and explicit measurement and visualization of the right and left heart output during this transition has not been previously accomplished. We combined 4D flow MRI (dynamic volumetric blood flow measurements) and T2 relaxometry (oxygen delivery quantification) in surgically instrumented newborn piglets to assess the DA. This was performed in Large White‐Landrace‐Duroc piglets (*n* = 34) spanning four age groups: term‐9 days, term‐3, term+1, and term+5. Subject's DA status was classified using 4D flow: closed DA, forward DA flow, reversed DA flow, and bidirectional DA flow. Over all states, vessel diameters and flows normalized to body weight increased with age (for example in the ascending aorta flow—term‐9: 126.6 ± 45.4; term+5: 260.2 ± 80.0 ml/min per kg; *p* = 0.0005; ascending aorta diameter—term‐9: 5.2 ± 0.8; term+5: 7.7 ± 0.4 mm; *p* = 0.0004). In subjects with reversed DA blood flow there was lower common carotid artery blood flow (forward: 37.5 ± 9.0; reversed: 20.0 ± 7.4 ml/min per kg; *p* = 0.032). Linear regression revealed that as net DA flow decreases, common carotid artery flow decreases (R^2^ = 0.32, *p* = 0.004), and left (R^2^ = 0.33, *p* = 0.003) and right (R^2^ = 0.34, *p* = 0.003) pulmonary artery flow increases. Bidirectional DA blood flow changed oxygen saturation as determined by MRI between the ascending and descending aorta (ascending aorta: 90.1% ± 8.4%; descending aorta: 75.6% ± 14.2%; *p* < 0.05). Expanded use of these techniques in preterm animal models will aid in providing new understandings of normal versus abnormal DA transition, as well as to test the effectiveness of related clinical interventions.

## Graphical abstract

At birth, the lungs and surrounding vasculature work to functionally close the ductus arteriosus (DA). This process is delayed in preterm newborns. MRI techniques, namely 4D flow and T2 relaxometry, can be used to comprehensively assess cardiac blood flow and oxygen delivery in newborns. These approaches are demonstrated across preterm and term‐born piglets, and whole heart blood flow visualization is paired alongside flow and oxygenation quantification.

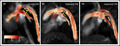

## 1 INTRODUCTION

Successful transition at birth is dependent on a coordinated and timely conversion from fetal circulation, in which the placenta acts as the organ of gas exchange, to postnatal circulation, in which that role is performed by the lungs. During fetal life, intra‐ and extra‐cardiac shunts ensure that oxygen‐ and nutrient‐rich blood is preferentially delivered to the brain and the heart. In optimal birth transition the key fetal extra‐cardiac shunt connecting the pulmonary and systemic circulation, the ductus arteriosus (DA), must achieve functional closure.

Closing of the DA shunt is caused by multiple inter‐related factors, including a rapid increase in partial pressure of oxygen (PaO_2_) as a consequence of lung aeration and the constricting action of oxygen. This leads to a rapid decrease of pulmonary vascular resistance and increase of pulmonary blood flow, as well as an increase of systemic vascular resistance induced by cord clamping (Hooper et al., 2016; Van Vonderen et al., 2014). Complex dynamic changes within the DA occur during this time; the fetal pulmonary‐to‐systemic (right‐to‐left) shunt flips direction, especially during low‐pressure ventricular diastole, to become left‐to‐right (systemic‐to‐pulmonary) shunting when averaged over the cardiac cycle (Clyman et al., 2012). These two patent DA states are complemented by a third, bidirectional DA flow, in which left‐to‐right flow occurs during systole and flips to right‐to‐left during diastole (Crossley et al., 2009). Functional closure of the DA takes approximately 1 to 3 days at term; however, in preterm neonates, DA closure is often delayed. This is in part a function of gestational age at birth; the more preterm, the more resistant the DA is to spontaneous closure. However, other factors including condition at birth, fetal growth restriction, and the availability of, or suitability for, interventions can influence DA closure rates (Sankar et al., 2019). Additionally, DA flow characteristics are important predictors of morbidity. Hemodynamically significant patent DA (hsPDA) is associated with reduced cerebral oxygenation and impaired perfusion of the kidneys and GI tract and the presence of pulmonary congestion with increased risk of ventilator dependence. These manifest clinically as increased risk of intraventricular hemorrhage, renal impairment and necrotizing enterocolitis, and bronchopulmonary dysplasia, respectively (Dice & Bhatia, 2007). With these clinical presentations, the hsPDA typically exhibits left‐to‐right blood shunting, termed “ductal steal,” in which retrograde flow occurs through the DA with increased blood flow into the pulmonary circuit but reduced flow into the systemic circuit.

Though these changes have been understood in human and animal models for several decades, the direct comprehensive visualization and quantification of the cardiac systemic and pulmonary output has not been reported during this transition. Coupled with well‐developed animal models of preterm and term neonates, advances in MRI technology, namely the abilities to measure whole‐chest blood flow using 4D flow MRI, “4D flow,” (Markl et al., 2012) and to quantify oxygen delivery in specific vessels using T2‐prepared MRI (Darby et al., 2020; Saini et al., 2020), allow for a more in‐depth probing of the dynamic and varied changes in DA flow characteristics occurring at birth.

The purpose of this study was to explore the changes in intracardiac and DA flow patterns that occur at birth by performing 4D flow MRI in a newborn piglet model at times when the DA is likely to be either functionally patent or not. This in vivo technique allows for comprehensive visualization and quantification of blood flow over a large imaging volume, in this case the entire piglet thorax. Quantitative and qualitative intracardiac blood flow pattern changes are investigated over a range of neonatal piglets born preterm or at term, in both the newborn period and at several days postnatal age with the aim of imaging a patent DA (as in fetal life) or a closed DA (as occurs soon after birth). These quantitative flow findings are validated against existing techniques and enhanced with the measurement of oxygenation estimation using MRI T2 relaxometry.

## 2 METHODS

### 2.1 Ethical approval and animal inclusion

All procedures were performed with approval from the South Australian Health and Medical Research Institute (SAHMRI) Animal Ethics Committee following the guidelines of the Australian Code of Practice for the Care and Use of Animals for Scientific Purposes set by the National Health and Medical Research Council (National Health and Medical Research Council, 2013). All investigators followed the ethical principles summarized by (Grundy, 2015) and complied with the ARRIVE guidelines (Kilkenny et al., 2010). Studies were performed using 14 Large White‐Landrace Cross sows mated with a Duroc boar from the Roseworthy Piggery (University of Adelaide, Australia). To generate preterm piglets, sows (average sow litter, *n* = 11) were housed at the Preclinical Imaging and Research Laboratories, SAHMRI from 84 days gestation (term, 115 days) in individual pens with ad libitum access to food and water. Term‐born piglets were derived from sows maintained at the Roseworthy piggery.

Thirty‐four neonatal piglets comprised of 17 males and 17 females were studied to investigate changes in cardiovascular flow patterns with age. Piglets were separated into four groups based on age when MRI was performed: term minus 9 days (T‐9; delivered by Cesarean section preterm at 106 days gestation and immediately scanned; time from cord cut to start of 4D flow = 49.1 ± 39.1 min; range = 20–140 min; *n* = 12; five males; seven females), term minus 3 (T‐3; delivered by Cesarean section and scanned at 112 days; time from cord cut to start of 4D flow = 38.2 ± 14.7 min; range =17–60 min; *n* = 10; six males; four females), term plus 1 (T+1; spontaneously delivered at 115 days and scanned within 12 h; *n* = 8; five males; three females), and term plus 5 (T+5; spontaneously delivered at 115 days and scanned at 120 days; *n* = 4; one male; three females). T+1 piglets were delivered spontaneously in good condition at the piggery, randomly selected from litters, and transported within 1 h in groups of 2–4 for imaging. As a result, scanning in the T+1 group was performed 6–12 h after spontaneous delivery. T+5 piglets were housed at the piggery with the sow for 5 days and transported to the MRI on the day of the study.

### 2.2 Preparation of piglets for MRI studies

For T‐9 and T‐3 piglets, pregnant gilts were fasted for a minimum of 12 h prior to being anesthetized. Pregnant gilts and newborn piglets were anesthetized with inhalation of isoflurane via a face mask and then administered an intramuscular ketamine injection (20 mg/kg), intubated, and maintained on 2%–3.5% isoflurane with an O_2_ and air mixture of ~2 L/min O_2_ and 4 L/min air. Gilts were placed supine on the surgical table and an abdominal incision was made to expose the uterus. The uterine horns were opened sequentially to expose each fetus.

For all piglet age groups, a vascular catheter was implanted in the carotid artery and jugular vein, and carotid arterial pressure was recorded (LabChart 7, ADInstruments). The piglet was intubated (uncuffed 2.5F endotracheal tube) and a blood sample was taken from the piglet carotid artery as a baseline blood gas measurement. In the T‐9 and T‐3 piglets, when all parameters were stable, the umbilical cord was clamped. All piglets were weighed and wrapped in a plastic bag for maintenance of piglet temperature as per standard neonatal intensive care practice before being immediately transferred to the MRI suite (3 Tesla clinical MRI system; MAGNETOM Skyra, Siemens Healthineers).

Piglets were placed on their left side in a custom‐built, MRI safe, heated cradle to maintain normothermia. The piglets were connected to a ventilator outside the magnet room via MRI‐compatible extension hoses. Anesthesia was maintained first with a bolus dose of propofol (5–10 mg, IV) and then continuously infused (5 mg/kg/h). To ensure balanced anesthesia, piglets received an IV bolus dose of fentanyl (5 µg/kg) and, to prevent movement while in the MRI, vecuronium (0.4 mg/kg). Neither betamethasone nor exogenous surfactant was required to achieve adequate lung compliance for stable postnatal transition in the T‐3, T+1, and T+5 piglets. T‐9 piglets received a 1.2 ml of bolus of Curosurf (Emerge Health) following intubation.

An MRI‐compatible oxygen saturation (SaO_2_) and heart rate monitor were applied to the piglet's ear for the duration of the scan (Nonin Medical Inc.) and data were recorded continuously using LabChart 7 (ADInstruments). The piglets were ventilated at a respiratory rate of 30–40 breaths per minute and fraction of inspired O_2_ titrated to achieve SaO_2_ = 90%–95% (peak inspiratory pressure = 22 cm H_2_O, positive end‐expiratory pressure = 5 cm H_2_O, tidal volume ~4 ml/kg). Due to issues with dead space and pressure degradation between the ventilator and the piglet, gas pressure and flow rates were titrated to ensure appropriate and stable chest wall movement prior to the commencement of the MRI scan sequences.

To monitor piglet health, vital signs were recorded and reviewed every 15 min during the scan. The carotid arterial catheter was connected to a displacement transducer and a quad‐bridge amplifier. The carotid arterial pressure waveform was recorded and processed in LabChart to generate a cardiac trigger with the interval between systolic peaks corresponding to the cardiac cycle length. Thus, cardiac‐resolved MRI could be achieved by collecting data and retrospectively sorting it based on the carotid pressure waveform (Schrauben et al., 2020; Schrauben, Saini, et al., 2019).

Piglet health was further monitored through the collection of carotid arterial blood samples during MRI. Measurement of whole blood arterial PaO_2_, partial pressure of carbon dioxide (PaCO_2_), pH, SaO_2_, hematocrit, hemoglobin, lactate, and glucose were measured with a RAPIDPOINT 500 (Siemens).

### 2.3 4D flow acquisition and processing

**FIGURE 1:**
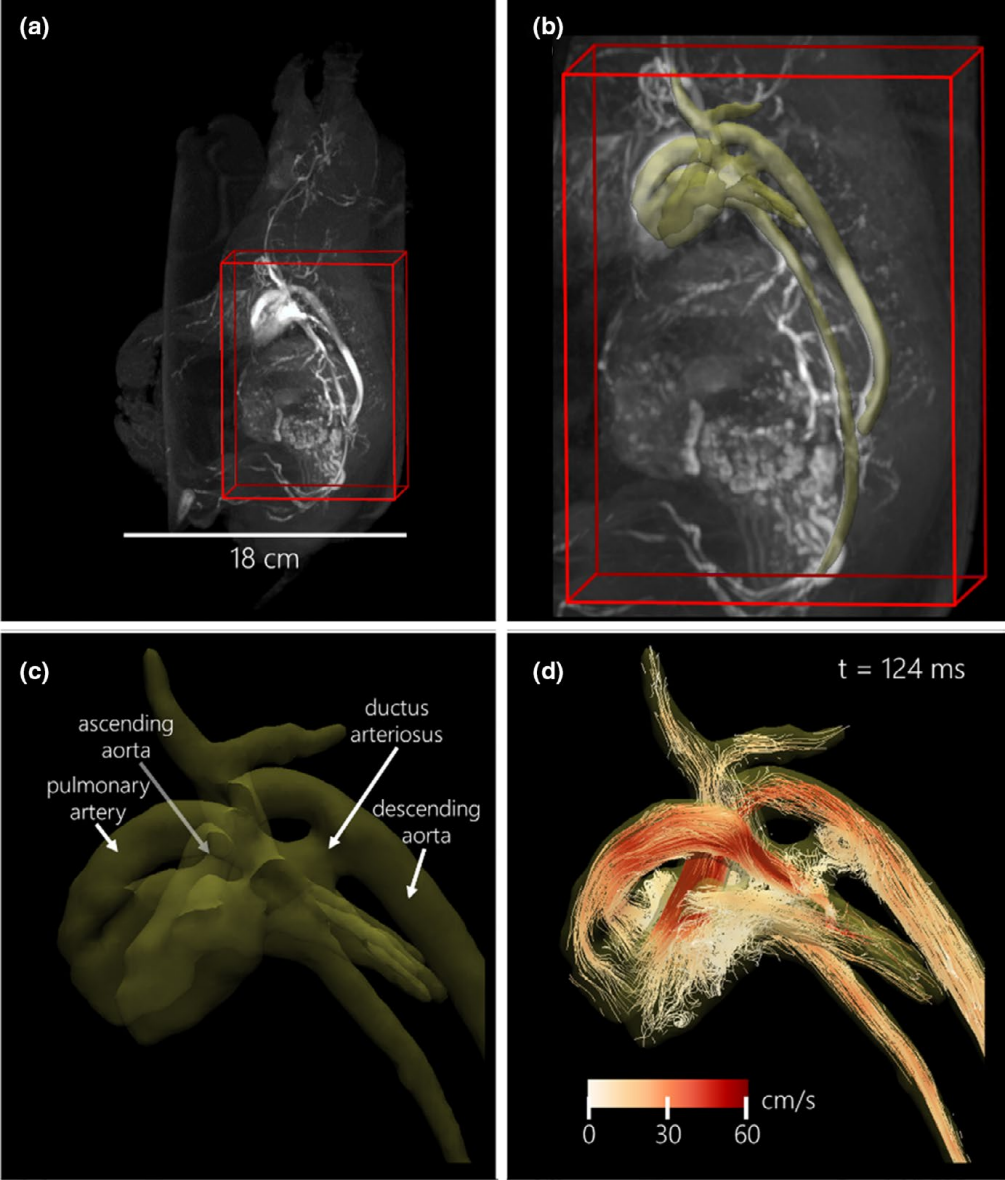
Example left lateral neonatal piglet images in a T‐3 1.4 kg male with open ductus arteriosus. (a) Whole piglet time‐of‐flight vascular imaging is used to prescribe the 4D flow acquisition (red wireframe). (b) Cardiac vasculature segmentation (yellow 3D rendering) is based on cardiac time‐averaged blood speed. (c) Zoomed view of labeled cardiac vessels of interest. (d) Particle trace visualization (color‐coded based on blood speed) at end‐systole (cardiac time = 124 ms)

Imaging was performed with a standard 16 channel cardiac receiver coil. Time‐of‐flight MRI angiography was used to visualize whole‐piglet vasculature. From these images, 4D flow was prescribed (Figure 1a) in an axial orientation for whole thoracic and abdominal coverage, using 138 ± 15 slices (depending on animal size). Acquired voxel size was 1 × 1 × 1 mm^3^, resulting in volumetric coverage of 176 × 176 × (138 ± 15) mm^3^. Specific 4D flow acquisition parameters over all subjects were: repetition time = 5.6 ms, echo time = 3.0 ms, and flip angle = 8°. The acquisition time, which depends on the piglet RR interval, was 12.8 ± 2.9 min. Velocity encoding, a parameter set in the 4D flow acquisition based on the highest expected blood velocity in the volume, was set to 70 cm/s for all subjects. All images were reconstructed to eight cardiac timeframes resulting in a temporal resolution of 52 ± 10 ms per timeframe (Schrauben et al., 2020; Schrauben, Saini, et al., 2019). The acquisition did not employ respiratory navigator gating or multiple averages.

Pre‐processing of 4D flow images utilized automated background phase correction and velocity unwrapping (Loecher et al., 2016). Corrected images (DICOM format) were then loaded into visualization and quantification analysis software (4D flow v2.4, Siemens) (Gulsun et al., 2012). Whole‐cardiac vasculature and surrounding great vessels were segmented directly from 4D flow data using time‐averaged velocity‐derived angiographic images (Figure 1b/c). Deformable registration was used to track the motion of vessels and update flow data over the cardiac cycle.

**FIGURE 2:**
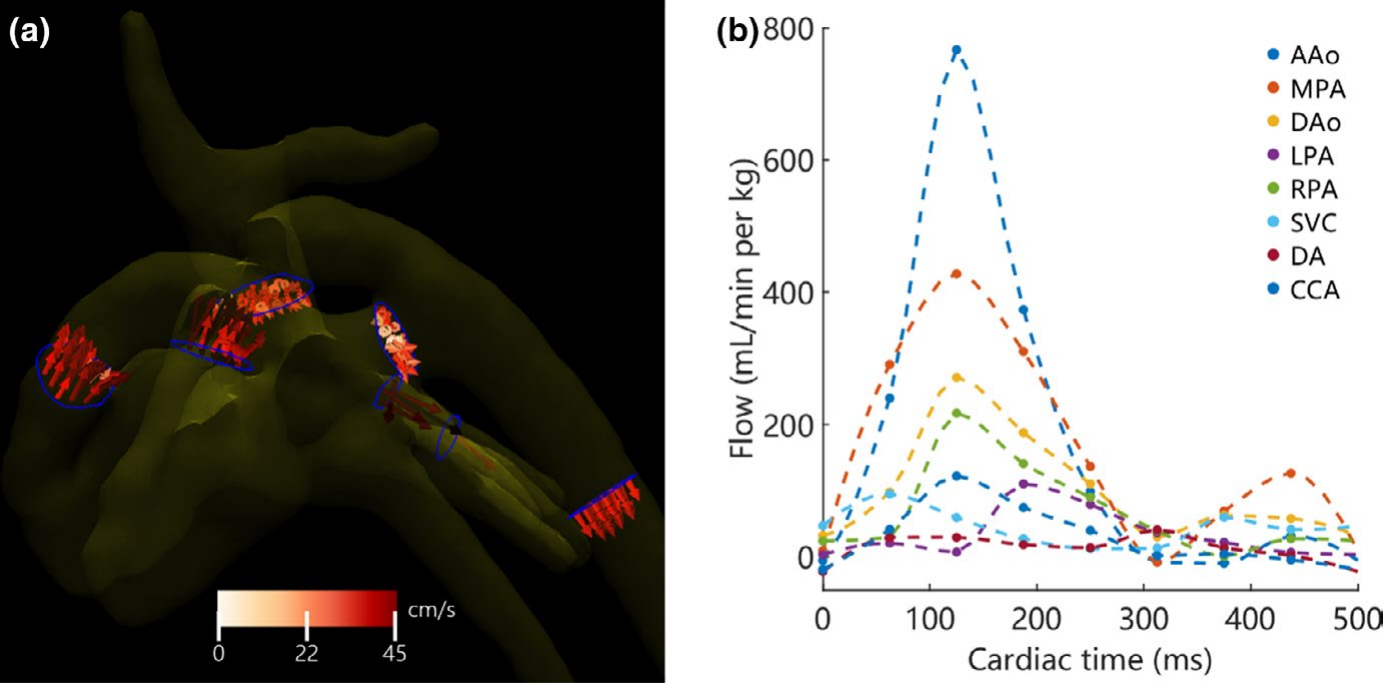
Example quantification results in the piglet from Figure 1. (a) Left lateral view of flow measurement contours (blue) in all vessels and 3D velocity vectors color‐coded to blood speed at peak systole (cardiac time = 124 ms). (b) The corresponding cardiac blood flow waveforms normalized to piglet weight for each measured vessel. A/D Ao, ascending/descending aorta; CCA, common carotid artery; SVC, superior vena cava; M/L/R PA, main/left/right pulmonary artery; DA, ductus arteriosus

4D flow measurements were taken in eight major cardiac vessels by automatic cross‐sectional contouring: the ascending and descending aorta (AAo, Dao—distal to the DA), the main, left, and right pulmonary arteries (MPA, LPA, RPA), the superior vena cava (SVC), the common carotid artery (CCA, or brachiocephalic artery), and the DA (if patent). Contours were manually inspected to ensure the capture of temporal changes in vessel cross‐sectional area and to ensure contours were perpendicular to the vessel and direction of flow (Figure 2). The calculated flow‐related parameters included cardiac cycle average flow normalized to fetal body weight (ml/min per kg) and cardiac cycle average vessel diameter (mm).

### 2.4 Validation of 4D flow measurements

Conservation of mass, which states that total blood flow must be conserved in a circuit, can be used as internal validation for 4D flow. Conservation of mass was assessed in systemic and pulmonary vasculature using the following equations (note that net DA flow can be positive or negative):

$$\text{MPA = LPA + RPA + DA}\;\left(\text{if patent}\right)\;\text{and AAo = SVC + DAo - DA}\;\left(\text{if patent}\right).$$

Additionally, 4D flow measurements of average flow over the cardiac cycle were compared against 2D PC MRI, which is considered the “gold standard” for MRI‐based flow measurements: in‐plane resolution = 1 × 1 mm^2^, slice thickness = 5 mm, repetition time = 5.6 ms, echo time = 3.2 ms, flip angle =30°, velocity encoding =70 cm/s, and 3 averages to increase image quality. The acquisition time per vessel was approximately 2.2 min. All images were reconstructed to 15 cardiac timeframes resulting in a temporal resolution of 27.7 ± 5.3 ms per timeframe. Measurements of 2D PC were not gathered in all vessels for reasons including scan time limitations, unresolved velocity aliasing, poor prescription, missing data, or DA not being visible.

### 2.5 Identification of the ductus arteriosus

At the small scale of vessels and intracardiac flow patterns in these piglets, identification of a patent DA is nontrivial. To identify and segment the DA, manually placed seed points were placed in the MPA and the DAo of the 4D flow data. A vessel‐tree centerline extraction was then performed using the analysis software. If the DA was patent, the MPA and DAo were connected by this centerline. Subjects were labeled as having a “closed DA” if this centerline did not connect the MPA and DAo; this does not negate the possibility that the DA flow was too low or the shunt too small for the 4D flow technique to detect. Once segmented, particle traces, which follow the time‐resolved paths of blood speed, were released from the MPA and DAo. These particles were used to determine DA blood direction at peak arterial systole and end‐diastole, as determined from the AAo flow waveform (Figure 2). This resulted in three patent DA states: (1) both systole and diastole were positive—forward or right‐to‐left shunting; (2) both systole and diastole were negative—reversed or left‐to‐right shunting; and (3) opposite direction between systole and diastole—bidirectional flow (Groves et al., 2008).

### 2.6 Determination of oxygen saturation

As an addition to blood gas information gained from the carotid artery catheter (tip in the ascending aorta) and MRI blood flow information, oxygen saturation was estimated in the AAo and DAo using MRI T2 relaxometry. The paramagnetic property of deoxyhemoglobin can be used to establish a relationship between the MRI time constant T2 of blood and blood SaO_2_ levels. Blood relaxometry was performed using a T2‐prepared pulse sequence with a balanced steady‐state free precession acquisition (MyoMaps, Siemens). MRI acquisition parameters over all subjects and vessels were: in‐plane resolution = 1.3 × 1.3 mm, slice thickness = 6 mm, repetition time = 4.2 ms, echo time = 2.1 ms, flip angle = 70°, T2 preparation times = [32, 64, 96, 128, 160, 192] ms, and acquisition time = 47 s. A non‐rigid motion correction algorithm was applied to compensate for in‐plane movement caused by piglet breathing.

The T2 relaxation time was analyzed in the AAo and DAo using custom lab‐developed software and as previously described (Saini et al., 2020). As the catheter was placed in the common carotid artery of the piglets, AAo SaO_2_ was expected to equal the values taken directly from the blood gas measurement during 4D flow scanning. Thus, with these two corresponding values, the Luz‐Meiboom exchange model (a quadratic equation) relating carotid SaO_2_ and AAo T2 relaxation time was derived (Luz & Meiboom, 1963; Saini et al., 2020). An additional eight in vitro samples taken from fetal pigs were utilized to better characterize the T2–SaO_2_ relationship. This relationship was then used to estimate SaO_2_ in the AAo and DAo from their respective T2 relaxation times.

### 2.7 Statistical analysis

Statistical testing was completed using R (R Core Team 2020, R Foundation for Statistical Computing; https://www.R‐project.org/). Data are presented as average ± standard deviation. Significance level for all testing was set to 5% (*p* < 0.05).

### 2.8 Validation of 4D flow measurements

Over all subjects and vessels where both techniques, 4D flow and 2D PC MRI, were successfully completed, least squares regression and Bland–Altman analysis were performed.

### 2.9 Age‐ and sex‐wise comparisons

Parameters used for statistical testing included animal characteristics, blood gas collection, and 4D flow parameters in each vessel. For the determination of differences across age, sex, and the interaction between age and sex, two‐way analysis of variance (ANOVA) with post hoc Tukey's test was used. As the T+5 age group only had one male, it was excluded for this two‐way ANOVA testing. Fewer animals were included in the T+5 group because the aim of the study was to observe an open or closed DA. Following this analysis, sex was collapsed, and one‐way ANOVA was performed to find differences across all age groups including T+5.

### 2.10 DA flow direction post hoc analysis

The direction of blood flow across the DA (here using DA direction at peak arterial systole and end‐diastole) determines shunting and may elucidate changes in neonatal health, particularly with oxygen delivery. This splits subjects into four groups based on DA status: closed, patent with forward flow (forward), patent with reversed flow (reversed), and patent with bidirectional flow. In piglets with a closed DA, differences in parameters measured for each age group were assessed using a one‐way ANOVA with post hoc Tukey's test. Note group numbers were insufficient for two‐way ANOVA with age and sex. In subjects with a patent (open) DA, differences between forward, reversed, and bidirectional DA flow were tested using two‐way ANOVA to examine the effects of DA flow direction, sex, and the interaction between the two. For this testing, separate age groups (T‐9, T‐3, and T+1) were collapsed. To further examine how the direction of DA flow affected surrounding vessels, least squares regressions of DA flow versus flow in each individual vessel were performed.

Finally, subjects that had AAo T2 measurements, DAo T2 measurements, or both were delineated by DA status. In subjects with no AAo T2 relaxometry, catheter SaO_2_ was used to increase group numbers. Note group numbers were insufficient for age and sex delineation and these factors were collapsed. These data were comprised of both paired and unpaired SaO_2_ values. However, the number of unpaired values was too small for an optimal pooled *t*‐test, and therefore an unpaired *t*‐test was performed to examine groupwise changes.

## 3 RESULTS

### 3.1 Group breakdown and animal blood gas status

Animal preparation and MRI scanning including 4D flow were completed in all 34 neonatal piglets, and no qualitative differences in image quality were observed between age groups.

There was a statistically significant increase in piglet weight, mean arterial blood pressure, and systolic blood pressure with age (Table 1). Among blood gas parameters, lactate was significantly lower in older piglets. There were no differences in any of the other measured blood gas parameters taken just before or during 4D flow acquisition. A large spread in piglet body temperature was observed for both the youngest and oldest groups.

**TABLE 1:** Piglet characteristics and blood gas parameters across age groups taken during MRI (mean ± standard deviation)

	Age relative to term (days)			
Characteristics	T−9	T−3	T+1	T+5
Sample (male:female)	5:7	6:4	5:3	1:3
Cord cut to start of 4D flow scan (min)	49.1 ± 39.1	38.2 ± 14.7	—	—
Weight (kg)	1.07 ± 0.14**abc**	1.59 ± 0.23	1.47 ± 0.47**f**	1.95 ± 0.16
Body temp during 4D flow (ºC)	34.0 ± 1.5**b**	35.0 ± 1.5	37.0 ± 0.4**f**	33.5 ± 2.4
Cardiovascular data				
Heart rate—4D flow (bpm)	153.8 ± 22.0	129.1 ± 19.7**e**	134.5 ± 20.3**f**	179.1 ± 32.2
Heart rate—catheter (bpm)	156.7 ± 24.8	132.1 ± 23.4**e**	145.4 ± 33.1	192.9 ± 45.5
Mean arterial pressure (mmHg)	47.1 ± 6.6**c**	53.3 ± 8.1	49.4 ± 13.6**f**	68.8 ± 17.1
Systolic blood pressure (mmHg)	55.2 ± 5.5**c**	62.5 ± 11.3**e**	63.6 ± 15.6**f**	94.1 ± 21.6
Diastolic blood pressure (mmHg)	43.1 ± 7.9	47.2 ± 7.6	42.4 ± 12.7	56.1 ± 15.3
Blood gas parameters				
PaO_2_ (mmHg)	223.8 ± 165.2	195.6 ± 97.2	276.3 ± 157.8	280.8 ± 38.7
PaCO_2_ (mmHg)	42.5 ± 19.0	53.9 ± 33.5	52.4 ± 27.2	32.2 ± 4.9
pH	7.451 ± 0.216	7.417 ± 0.250	7.358 ± 0.224	7.444 ± 0.078
SaO_2_ (%)	93.2 ± 7.4	95.0 ± 3.8	93.0 ± 6.8	93.0 ± 8.6
Hematocrit (%)	26.9 ± 3.0	24.2 ± 4.2	25.8 ± 2.2	21.3 ± 4.9
Hemoglobin (g/L)	91.6 ± 9.9	82.0 ± 14.6	87.5 ± 8.3	71.8 ± 16.5
Lactate (mmol/L)	4.13 ± 1.68**bc**	4.49 ± 1.67**de**	1.75 ± 0.98	0.78 ± 0.27
Glucose (mmol/L)	4.45 ± 1.87	3.98 ± 1.90	6.02 ± 1.29	5.90 ± 1.49

Parameter differences across age groups were tested using one‐way ANOVA. Inter‐group significant differences are labeled: **a**: T‐9 versus T‐3; **b**: T‐9 versus T+1; **c**: T‐9 versus T+5; **d**: T‐3 versus T+1; **e**: T‐3 versus T+5; **f**: T+1 versus T+5.

### 3.2 Visualization of intracardiac flows

**FIGURE 3:**
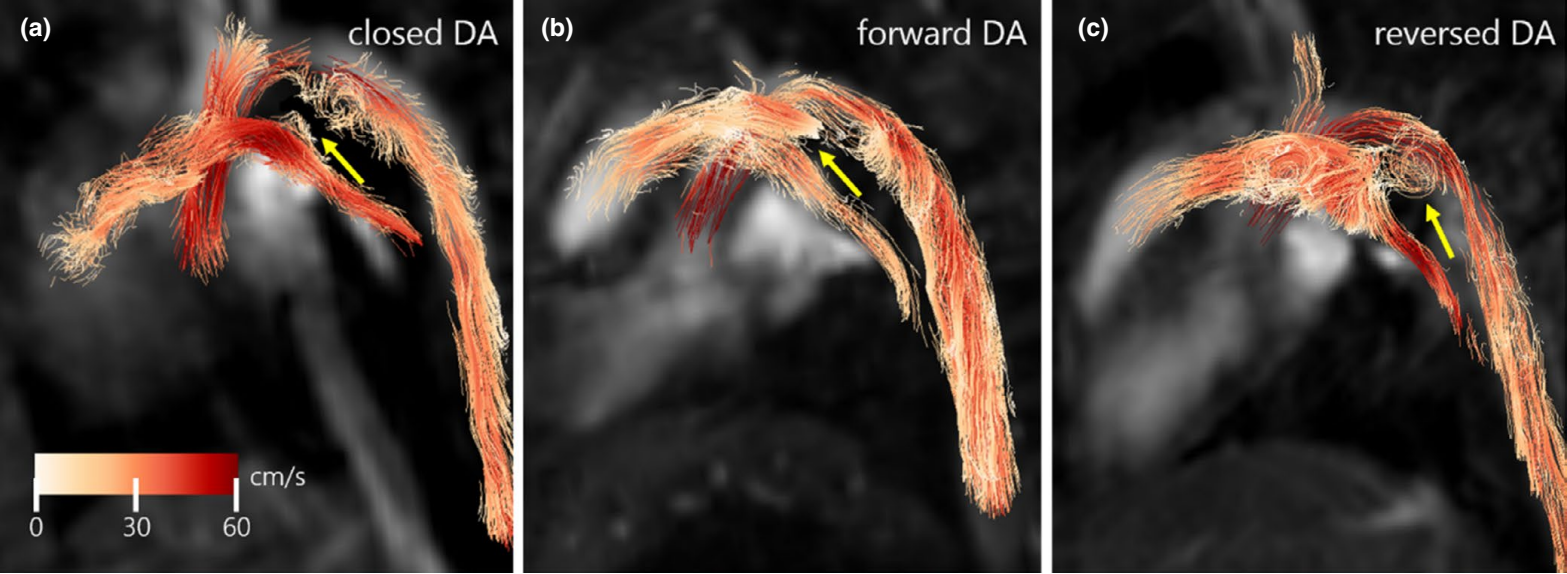
Example peak systolic flow particle trace visualizations of piglets in a left lateral view with closed DA (a, T‐9, 0.99 kg male), forward DA flow (b, T‐3, 1.48 kg male), and reversed DA flow (c, T‐3, 1.80 kg male). DA location is denoted by yellow arrows. Note swirling flow and vortex formation in the DA in (c). Animated versions of these particle trace visualizations can be found in Video S1, Video S2, and Video S3. DA, ductus arteriosus

Example particle trace visualizations of cardiac blood flow at peak arterial systole to determine DA status are shown in three piglets (Figure 3; **a**: T‐9, 0.99 kg male; **b**: T‐3, 1.48 kg male; **c**: T‐3, 1.80 kg male). Corresponding animated versions of each panel can be found in Video S1, Video S2, and Video S3. The DA status and flow direction are labeled as closed with an apparent lack of flow (Figure 3a), patent and forward (right‐to‐left, Figure 3b), and patent and reversed (left‐to‐right, Figure 3c).

### 3.3 Validation of 4D flow measurements

Conservation of mass using 4D flow was calculated in all subjects (*n* = 34 measurements). This resulted in high internal consistency, with the percent difference averaged over all subjects (mean ± standard deviation): systemic = 6.21% ± 6.04% and pulmonary = 8.70% ± 5.46%.

**FIGURE 4:**
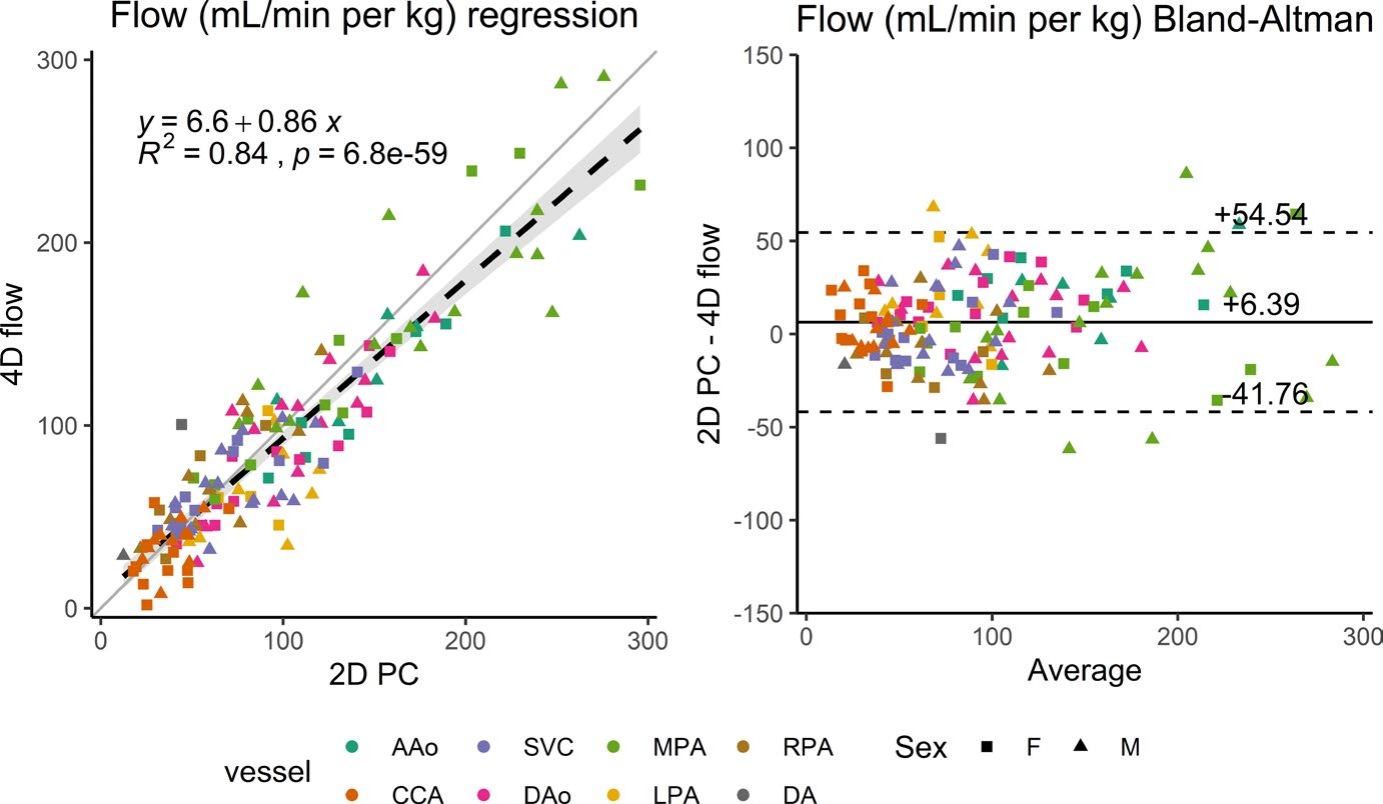
2D PC MRI versus 4D flow vessel by vessel comparison of blood flow (ml/min per kg) using linear regression (left) and Bland–Altman analysis (right). For regression, the best fit line (dashed black) is shown with 95% confidence intervals (gray shading), as well as the unity line (gray) for reference. A/D Ao, ascending/descending aorta; CCA, common carotid artery; SVC, superior vena cava; M/L/R PA, main/left/right pulmonary artery; DA, ductus arteriosus

In the 34 piglets, 2D PC MRI of different vessels was successfully performed in the following proportions: AAo =13/34 (38%), MPA = 28/34 (82%), DAo = 27/34 (79%), LPA = 14/34 (41%), RPA = 15/34 (44%), SVC = 26/34, (76%), CCA = 22/34 (65%), and DA = 2/24 (8%). This resulted in 147 direct flow measurement comparisons between 2D PC MRI and 4D flow (Figure 4). Good correlation between the techniques (R^2^ = 0.84, slope = 0.86, 95% confidence interval [0.80, 0.93]) was observed, resulting in a bias of +6.39 ml/min per kg (limits of agreement = [−41.8, 54.5]).

### 3.4 Identification of the ductus arteriosus

While the DA was fully closed in all T+5 subjects (4/4, 100%), it was open in some proportion in the three other age groups (Table 2): T‐9 (9/12, 75%), T‐3 (10/10, 100%), and T+1 (5/8, 63%). Among subjects with patent DA, forward, reversed, and bidirectional DA flow was observed in the following proportions: T‐9 (forward—2/9, 22%, reversed—4/9, 44%, bidirectional—3/9, 33%), T‐3 (forward—3/10, 30%, reversed—5/10, 50%; bidirectional—2/10, 20%), and T+1 (forward—1/5, 20%, reversed—0/5, 0%; bidirectional—4/5, 80%).

**TABLE 2:** DA patency status and flow direction in piglets with an open DA as determined by 4D flow. DA flow direction was determined from peak arterial systole and end‐diastole

	Age relative to term (days)			
DA status	T−9	T−3	T+1	T+5
Closed	3	0	3	4
Open	9 (75%)	10 (100%)	5 (63%)	0 (0%)
Forward	2	3	1	—
Reversed	4	5	0	—
Bidirectional	3	2	4	—

### 3.5 Effect of age and sex on blood flow measurements

**FIGURE 5:**
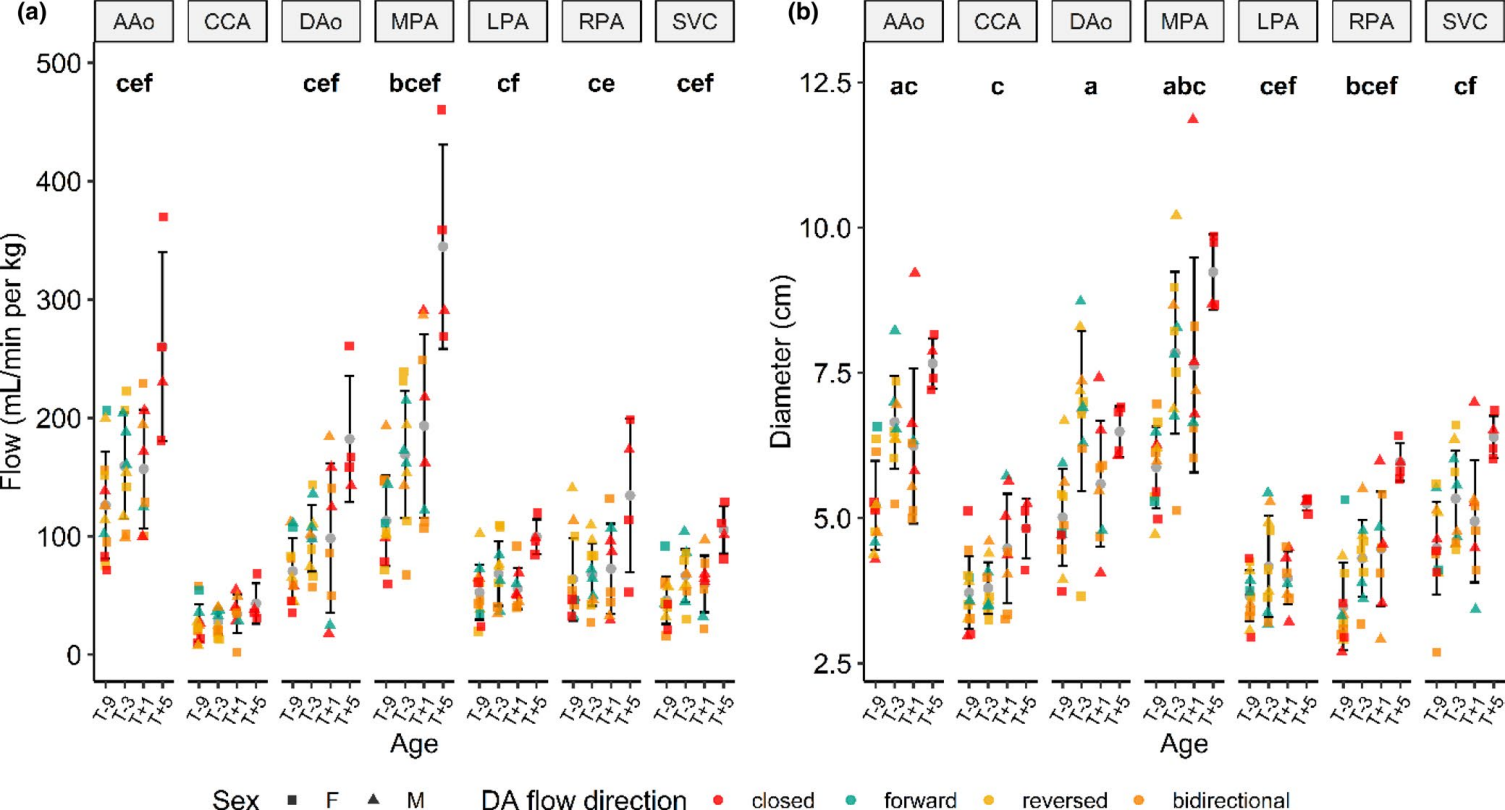
4D flow blood flow (left, in ml/min per kg) and diameter (right, in mm) measurements (mean ± standard deviation) across all major cardiac vessels and piglets, delineated by gestational age, sex (shapes), and direction of DA blood flow (colors: closed, forward, reversed, bidirectional). Within each age group, differences are statistically tested using one‐way ANOVA and shown at the top of each plot: **a**: T‐9 versus T‐3; **b**: T‐9 versus T+1; **c**: T‐9 versus T+5; **d**: T‐3 versus T+1; **e**: T‐3 versus T+5; **f**: T+1 versus T+5. A/D Ao, ascending/descending aorta; CCA, common carotid artery; SVC, superior vena cava; M/L/R PA, main/left/right pulmonary artery

Summary results for blood flow and diameter across all vessels and piglets are shown in Figure 5. These are delineated by the four gestational age groups. From two‐way ANOVA, there was no effect of sex on 4D flow parameters from T‐9 to T+1 and no interaction between the effect of sex and age (3 ages).

From the statistical comparisons using one‐way ANOVA across ages, all flows except the CCA increased from T‐9 to T+5, while the AAo, DAo, and MPA increased from both T‐3 to T+5 and T+1 to T+5. This increase in flow is preceded by an earlier increase in diameter in all vessels from preterm to postnatal.

### 3.6 DA status post hoc analysis

**FIGURE 6:**
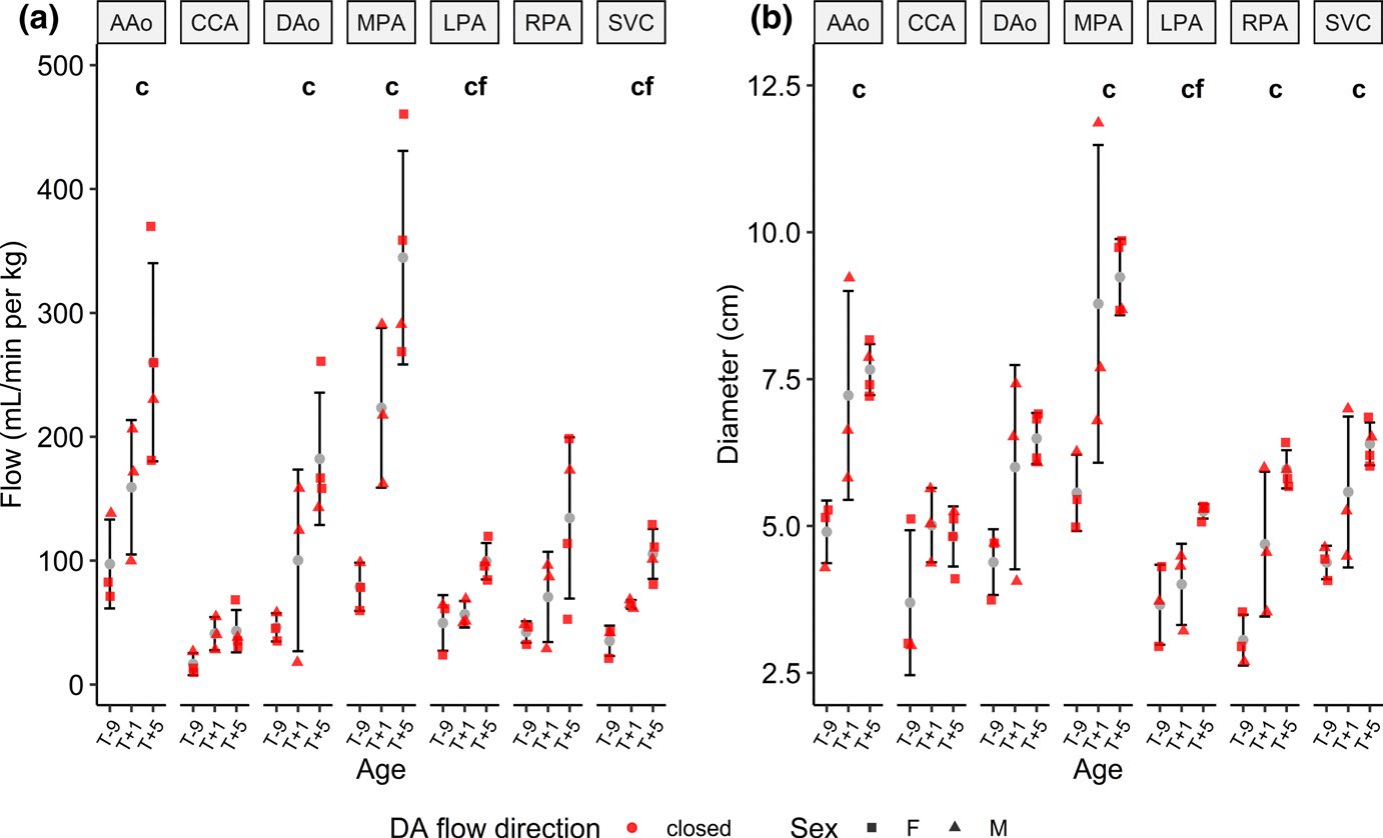
4D flow blood flow (left, in ml/min per kg) and diameter (right, in mm) measurements (mean ± standard deviation) across age in piglets with a closed ductus arteriosus. Differences were assessed using one‐way ANOVA for each vessel: **c**: T‐9 versus T+5; **f**: T+1 versus T+5. A/D Ao, ascending/descending aorta; CCA, common carotid artery; SVC, superior vena cava; M/L/R PA, main/left/right pulmonary artery

Using one‐way ANOVA in piglets with a closed DA (Figure 6), significantly lower blood flow was observed in T‐9 compared to T+5 piglets (AAo, MPA, DAo, LPA, and SVC), while in the same groups, diameter was significantly lower in the AAo, MPA, LPA, RPA, and SVC. Among piglet characteristics, cardiovascular data, and blood gas data, weight (T‐9 vs. T+1; T‐9 vs. T+5) increased with age, while lactate (T‐9 vs. T+5) decreased.

**FIGURE 7:**
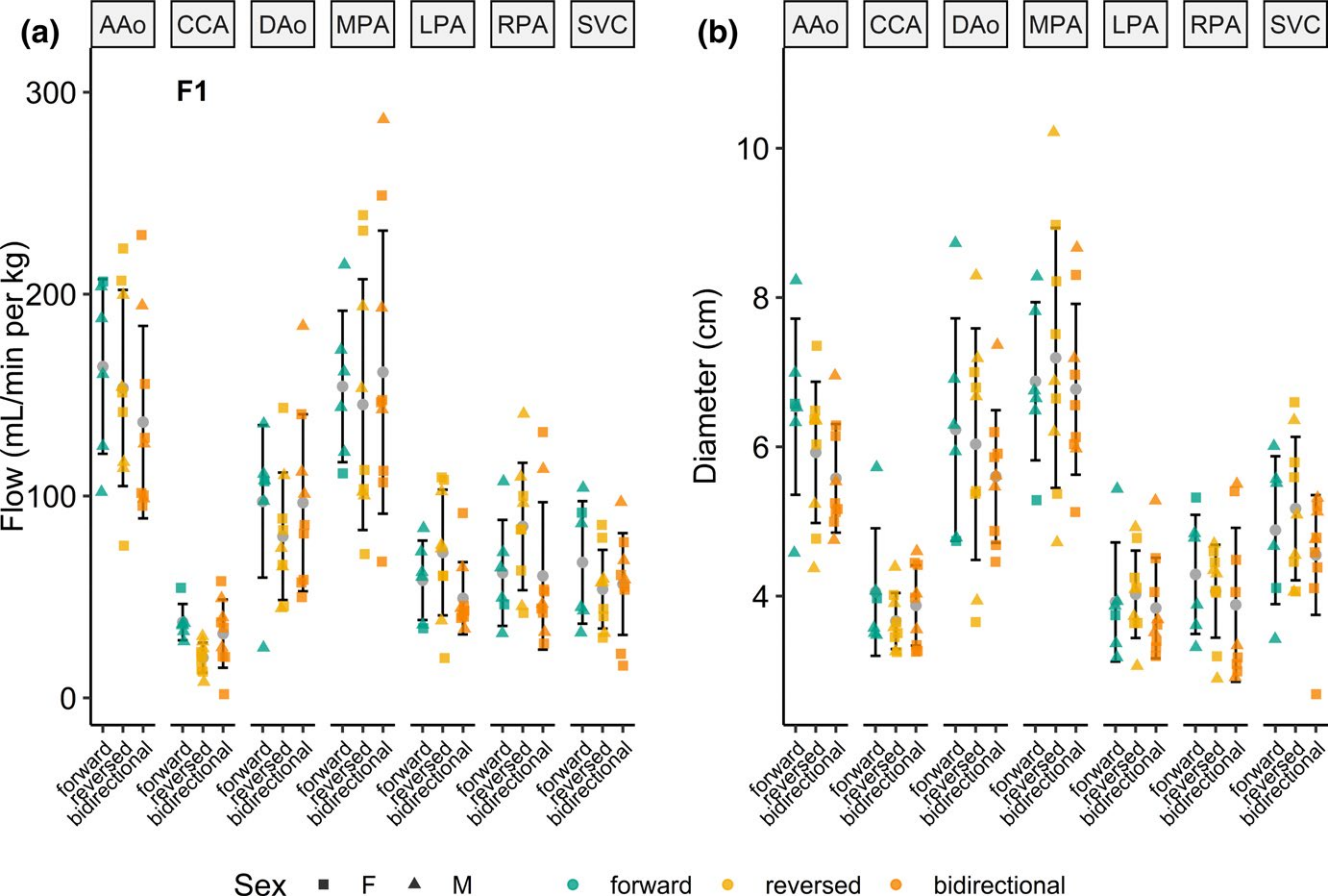
4D flow blood flow (left, in ml/min per kg) and diameter (right, in mm) measurements (mean ± standard deviation) across age in piglets with a patent ductus arteriosus (DA), delineated by sex (shapes) and direction of DA blood flow. Using two‐way ANOVA, a reversed DA significantly lowered CCA flow compared with forward DA (**F1**). No significant differences were observed across all other vessels and DA flow directions. A/D Ao, ascending/descending aorta; CCA, common carotid artery; SVC, superior vena cava; M/L/R PA, main/left/right pulmonary artery

Patent DA blood flow and diameter comparisons were tested using two‐way ANOVA (effects: DA blood flow direction and sex) and are shown in Figure 7. Compared with forward DA flow, statistically significantly lower CCA blood flow in subjects with reversed DA blood flow was observed, with no effects of sex or the interaction of DA flow direction and sex. No significant differences were observed for blood flow or diameter across all remaining vessels and DA flow directions. For piglet characteristics, cardiovascular data, and blood gas data, no statistically significant differences were observed over DA flow direction or sex (or their interaction).

**FIGURE 8:**
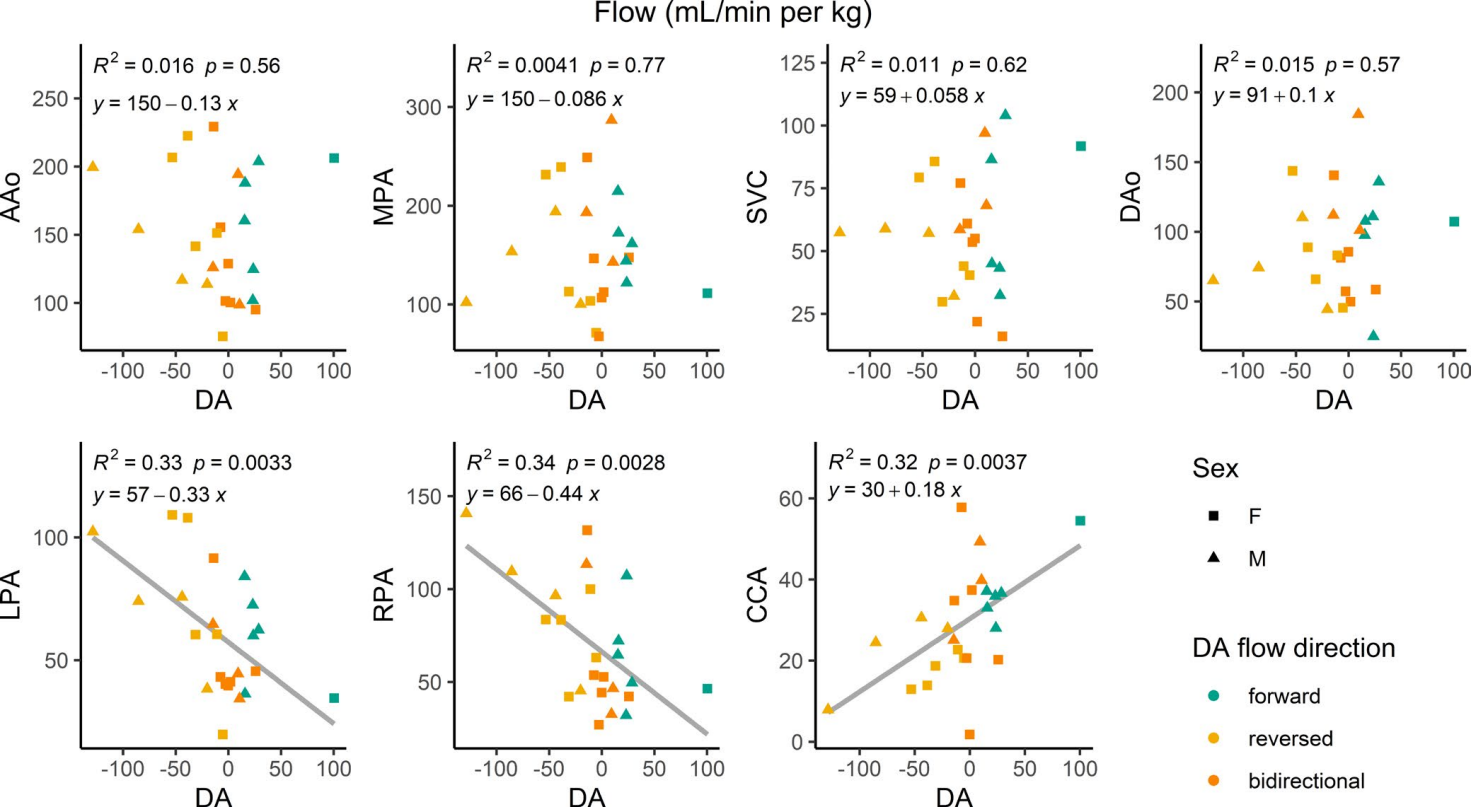
4D flow linear regressions of ductus arteriosus (DA) blood flow versus flow in surrounding vessels (in ml/min per kg), delineated by sex (shapes) and direction of DA blood flow. A/D Ao, ascending/descending aorta; CCA, common carotid artery; SVC, superior vena cava; M/L/R PA, main/left/right pulmonary artery

Least‐squares regressions of patent DA flow versus flow in surrounding vessels are shown in Figure 8. No significant correlation was observed between DA flow and AAo, DAo, MPA, or SVC flow. As DA flow decreases (more left to right shunting), significant decreases occur in CCA flow (R^2^ = 0.32, *p* = 0.004), while LPA (R^2^ = 0.33, *p* = 0.003) and RPA (R^2^ = 0.34, *p* = 0.003) flow are significantly increased.

### 3.7 Impact of DA patency on DAo oxygen saturation

Vessel T2 relaxometry imaging was successfully performed and analyzed in the following proportions: T‐9 (AAo = 11/12, 92%; DAo =11/12, 92%), T‐3 (AAo = 3/10, 30%; Dao = 1/10, 10%), T+1 (AAo = 7/8, 88%; Dao = 7/8, 88%), T+5 (AAo = 2/4, 50%; Dao = 2/4, 50%). Combining the 23 AAo T2 and eight additional in vitro T2 measurements allowed for 31 values to be used in the fitting of the T2–SaO_2_ relationship curve. This resulted in fit parameters: $T_{2,0}$ = 175.5 ms, K0 = 0.00402 ms^−1^, K1 = 0.01649 ms^−1^ (R^2^ = 0.72). This curve was then used to calculate the SaO_2_ in blood vessels where T2 relaxation times were measured.

**FIGURE 9:**
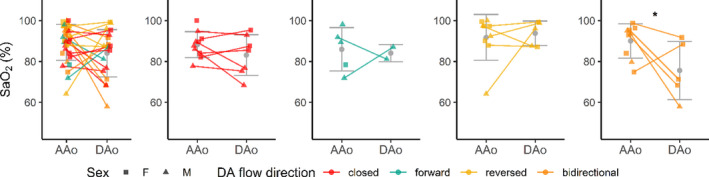
T2 MRI relaxometry‐based AAo SaO_2_ estimates versus DAo SaO_2_ estimates (%), grouped by DA flow direction. As there were not enough unpaired measurements for optimal pooled *t*‐test, unpaired t‐test was performed with age and sex collapsed (*p* < 0.05). Only “bidirectional” DA flow showed a statistically significantly higher AAo SaO_2_. DAo, descending aorta; DA, ductus arteriosus

Figure 9 displays T2 MRI estimated SaO_2_ values in the AAo compared with those estimated in the DAo, delineated by DA flow direction and sex. Intra‐subject values are connected. Among all data, significant differences (*p* < 0.05) were observed between AAo and DAo SaO_2_ values only in the “bidirectional” DA flow group. Group breakdowns of SaO_2_ were as follows: all grouped together—AAo (*n* = 33) =89.4% ± 8.9% versus DAo (*n* = 19) =84.1% ± 11.6%; closed—AAo (*n* = 10) =88.3% ± 6.4% versus DAo (*n* = 7) =83.2% ± 9.9%; forward—AAo (*n* = 5) =85.9% ± 10.6% versus DAo (*n* = 2) =84.1% ± 4.2%; reversed—AAo (*n* = 9) =91.8 ± 11.2% versus DAo (*n* = 5) =93.8% ± 6.0%; and bidirectional—AAo (*n* = 9) =90.1% ± 8.4% versus DAo (*n* = 5) =75.6% ± 14.2%.

## 4 DISCUSSION

Here we presented the assessment of DA and great vessel blood flow, blood gas measurements, and MRI‐derived SaO_2_ in a cohort of 34 neonatal piglets at different ages, spanning those born and studied preterm, at term, and several days old. DA identification and status were determined with 4D flow segmentation and visualization in order to classify the DA as closed, patent with forward flow, patent with reversed flow, or patent with bidirectional flow. Combined with different ages, this information provides a novel and comprehensive investigation of the role of DA blood flow in early life.

4D flow validation results had good overall internal agreement (systemic = 6.2%, pulmonary = 8.7%). Compared with previous 4D flow animal studies, namely in fetal sheep with cardiac and hepatic vessels of similar size (Schrauben et al., 2020; Schrauben, Saini, et al., 2019), internal consistency was improved (cardiac: 7.4%–18.8%, hepatic: 15.3%). A good correlation between 4D flow and 2D PC MRI and an underestimation of 4D flow values were observed. While 2D PC MRI is the gold standard for flow measurements with MRI in a single location, the difficulty of planning each slice, the acquisition time per slice (~2 min), and the relatively thick slices (5 mm) can be mitigated using 4D flow. Namely, the isotropic resolution of 4D flow, combined with the high internal consistency and ability to retrospectively place measurement planes in any orientation, compel its use in newborn piglets.

Regardless of the patency of the DA, there was an increase in body weight, heart rate, and mean and systolic blood pressure with increasing age. Similarly, cardiac output as measured by 4D flow significantly increased with age. Interestingly, this increase in cardiac output observed with age was preceded by an increase in vessel diameter, usually in the previous age group. Despite a significant increase in body weight from T‐9 to T+5 (from ~1 kg to ~2 kg), blood flow normalized to body weight was significantly higher in the T+5 subjects for all vessels. This suggests that in the first 5 days after birth, pulmonary and systemic demand is increased and may be met by an increase in heart rate rather than stroke volume (Giraud et al., 1995). This is further supported by increases in blood pressure with age.

In the detection of a patent DA using 4D flow, an unexpected result was the increased detection of open DA in T‐3 subjects (10/10) versus T‐9 subjects (9/12). This is possibly a result of using the same acquisition parameters for 4D flow across piglets of all sizes and should be considered a limitation of this study. While scans were performed at relatively high spatial resolution (1 × 1 × 1 mm^3^), the likely smaller caliber DA, coupled lower blood flow, in T‐9 subjects may render it unidentifiable at this resolution. These potentially undetectably small vessels are reflected by the smaller average DAo vessel diameter in the T‐9 piglets compared with the T‐3 piglets (5.0 ± 0.8 mm vs. 6.8 ± 1.4 mm). In older piglets, the DA was more likely to be closed (T‐9 = 3/12, T‐3 = 0/10, T+1 = 3/8, and T+5 = 4/4). The post hoc closed DA testing of 4D flow parameters revealed lower flow and diameter in the T‐9 group, following results over all piglet ages and DA statuses, as listed above.

Over subjects with a patent DA, post hoc analysis of DA flow direction with age collapsed was performed yielding statistically significant changes only in the CCA, with higher blood flow when DA flow was forward (right‐to‐left) versus reversed (left‐to‐right). As CCA diameter was unchanged in these two states, these results provide evidence of upper body and brain “ductal steal” in the case of reversed DA flow. Regressions between flow in the DA and flow in all other vessels demonstrate this effect where ductal steal decreases CCA flow while increasing flow in the branch pulmonary arteries (LPA and RPA) as a function of decreasing (more negative) DA flow. The dynamic nature of DA flow after birth may be difficult to capture in a 12 min 4D flow acquisition, especially in the consideration of the dependence of DA flow on systemic to pulmonary pressure gradients (Crossley et al., 2009).

The implications of diminished cerebral blood flow are important. Previous preterm human ultrasound data suggest that systemic hypoperfusion (manifesting as low superior vena cava blood flow) resulting from DA shunting is strongly associated with late intraventricular hemorrhage (Kluckow & Evans, 2000). Systemic hypoperfusion in the presence of a patent DA has also been demonstrated using 2D PC MRI, yet direct DA measurements were not made (Broadhouse et al., 2013). Recent work has suggested that early routine treatment to close the DA may not be necessary (Clyman et al., 2019) and current clinical practice is widely varied. However, the longer‐term consequences of reduced cerebral perfusion or oxygenation due to patent DA with “ductal steal” on brain development are unknown. Unless contraindicated, early ductal closure may be more feasible with the recent advent of patent DA occlusion by catheter intervention in preterm infants. Indices that are used to determine hsPDA are traditionally determined by ultrasound (Shepherd & Noori, 2019). However, robust diagnostic criteria for intervention and optimal medical treatment for hsPDA remain to be established (Sung, 2019). The combination of whole‐heart flow quantification using 4D flow and T2 relaxometry MRI in major blood vessels (ascending and descending aorta) renders a comprehensive representation of the changes occurring in the newborn period and may help to better understand optimal transition patterns in preterm and term neonates. The translation of this knowledge to clinical care will enable clinicians to understand the physiological consequences of ductal patency and its flow patterns. This will in turn inform clinical decision making around medical intervention to promote ductal closure; as no medication or intervention is without side effects in this patient group, understanding the safety of treatment versus permissive tolerance of ductal patency is key.

Finally, T2 relaxometry within the AAo and DAo, coupled with SaO_2_ measurements from the carotid catheter taken during 4D flow allowed for the calculation of a novel blood T2–SaO_2_ relationship in piglets. From this relationship, estimated DAo SaO_2_ values allowed for a post hoc analysis of DA flow direction and changes in SaO_2_ from the AAo (carotid SaO_2_) to the DAo. Only bidirectional DA flow showed a statistically higher AAo SaO_2_ compared with estimated DAo SaO_2_.

### 4.1 Limitations

A few limitations need to be mentioned. First, while the piglets were ventilated at a constant rate of 30–40 breaths/min to produce stable chest wall movement, no respiratory navigator gating was used in the 4D flow acquisition. No obvious respiratory motion artifacts were observed in the reconstructed images, yet the small scale of the vessels measured here may be affected by subtle motion, particularly the LPA and RPA. Respiration could be mitigated with the planning of respiratory navigators but would come at the cost of longer acquisition times.

Second, this study only examined large cardiac output vessels (and SVC) using 4D flow. Though not performed here, measurements of cardiac input, including the inferior vena cava and foramen ovale shunting (if present) would further elucidate the function of the DA in early life.

Third, the T2–SaO_2_ relationship may not be optimal. The technique is based on previous work from our lab (Saini et al. 2020). In that study, the T2–SaO_2_ curve was derived using in vitro blood drawn from both fetal and maternal sheep in four different vessels (arteries and veins). This allowed for a wide range of SaO_2_ (10%–100%) over 73 total blood samples. By comparison, the combined in vitro and in vivo calibration presented here only used 33 total blood samples, comprising of a smaller range of SaO_2_ values (20%–100%). Notably, a majority of the blood samples (22/33, 67%) had an SaO_2_ > 90%. These factors likely introduced more inaccuracies into our model fit (previous work in sheep R^2^ = 0.93; piglets R^2^ = 0.72); further validation of the pig blood T2–SaO_2_ relationship is required for future use, particularly if the range of SaO_2_ is below the physiological normal for adults or aims to measure SaO_2_ in the fetus.

The use of anesthesia in a surgically prepared preterm animal model allows for the implementation of the relatively long scan times (>10 min) needed to acquire high‐resolution 4D flow and to reduce movement artifacts. While the use of 4D flow to examine DA patency and characteristics has been reported in a single‐case study of a preterm infant (Broadhouse et al., 2015), broad application in this population is limited by technical challenges. These include logistical constraints (e.g., transfer to MRI suite, high image resolution, scan time, physiological challenges of maintaining body temperature and vital signs monitoring, and control of movement artifact) involved in scanning extremely preterm infants who are the patient group most likely to need DA treatment yet are the most clinically compromised. Work is ongoing to address these challenges in both human fetal and neonatal MRI (Goolaub et al., 2018; Roberts et al., 2020; Schrauben, Lim, et al., 2019).

Finally, it is tantalizing to consider using this approach to capture the actual process of changes in the DA at birth. For this to be feasible, the sow would need to be in the scanner with the intubated fetus positioned on her abdomen for localization scans. Ventilation, cord clamping, and the start of 4D flow would need to happen nearly simultaneously. However, a 200 kg sow would put the fetus above the optimal scanning plane in the bore. Additionally, the sequence runs for roughly 12 min, creating an average flow in each vessel over that time. Thus, minute by minute or second by second changes in flow in the DA cannot be measured using this approach, as can be done using flow probes. The advantage of 4D flow is that quantification and visualization can be performed in multiple vessels.

## 5 CONCLUSION

In conclusion, the optimal therapeutic goal for persistent DA flow in preterm infants remains uncertain. Defining vascular biomechanics in clinically relevant translational models of human preterm birth will provide novel insights and ultimately a platform for clinical innovation. The techniques presented here could be employed to determine normal DA transition timing and to test the efficacy of agents in opening or closing the DA.

## CONFLICT OF INTERESTS

The authors have no competing interests to disclose.

## AUTHOR CONTRIBUTIONS

Study Design: EMS, JRTD, MJB, MS, CKM, JLM. Conducting experiments: JRTD, MJB, BSS, MQ, SLH, ELB, MCL, JLM. Data Acquisition: JRTD, MJB, BSS, SLH, ELB, MCL, JLM. Data Analysis: EMS, JRTD, MQ, SLH, ELB, SKSC, TA, JLM. Manuscript writing: EMS, JRTD, MJB, BSS, MS, CKM, JLM. All authors approved the final version of the manuscript, agree to be accountable for all aspects of the work in ensuring that questions related to the accuracy or integrity of any part of the work are appropriately investigated and resolved. All persons designated as authors qualify for authorship, and all those who qualify for authorship are listed.

## Supporting information



Video S1

Video S2

Video S3

## Data Availability

The data that support the findings of this study are available from the corresponding author upon reasonable request.

## References

[phy214999-bib-0001] Broadhouse, K. M., Price, A. N., Durighel, G., Cox, D. J., Finnemore, A. E., David Edwards, A., Hajnal, J. V., & Groves, A. M. (2013). Assessment of PDA shunt and systemic blood flow in newborns using cardiac MRI. NMR in Biomedicine, 26(9), 1135–1141. 10.1002/nbm.2927 23412748

[phy214999-bib-0002] Broadhouse, K. M., Price, A. N., Finnemore, A. E., Cox, D. J., David Edwards, A., Hajnal, J. V., & Groves, A. M. (2015). 4D Phase contrast MRI in the preterm infant: Visualisation of patent ductus arteriosus. Archives of Disease in Childhood: Fetal and Neonatal Edition, 100(2), F164. 10.1136/archdischild-2013-305281 24907162

[phy214999-bib-0003] Clyman, R. I., Couto, J., & Murphy, G. M. (2012). Patent ductus arteriosus: Are current neonatal treatment options better or worse than no treatment at all? Seminars in Perinatology, 36(2), 123–129. 10.1053/j.semperi.2011.09.022 22414883 PMC3305915

[phy214999-bib-0004] Clyman, R. I., Liebowitz, M., Kaempf, J., Erdeve, O., Bulbul, A., Håkansson, S., Lindqvist, J., Farooqi, A., Katheria, A., Sauberan, J., Singh, J., Nelson, K., Wickremasinghe, A., Dong, L., Hassinger, D. C., Aucott, S. W., Hayashi, M., Heuchan, A. M., Carey, W. A., … Sun, Y. (2019). PDA‐TOLERATE trial: An exploratory randomized controlled trial of treatment of moderate‐to‐large patent ductus arteriosus at 1 week of age. Journal of Pediatrics, 205, 41–48.e6. 10.1016/j.jpeds.2018.09.012 PMC6502709 30340932

[phy214999-bib-0005] Crossley, K. J., Allison, B. J., Polglase, G. R., Morley, C. J., Davis, P. G., & Hooper, S. B. (2009). Dynamic changes in the direction of blood flow through the ductus arteriosus at birth. Journal of Physiology, 587(19), 4695–4704. 10.1113/jphysiol.2009.174870 PMC2768022 19675069

[phy214999-bib-0006] Darby, J. R. T., Schrauben, E. M., Saini, B. S., Holman, S. L., Perumal, S. R., Seed, M., Macgowan, C. K., & Morrison, J. L. 2020. Umbilical vein infusion of prostaglandin I2 increases ductus venosus shunting of oxygen rich blood but does not increase cerebral oxygen delivery in the fetal sheep. The Journal of Physiology, 598(21), 4957–4967. 10.1113/JP280019 32776527

[phy214999-bib-0007] Dice, J. E., & Bhatia, J. (2007). Patent ductus arteriosus: An overview. The Journal of Pediatric Pharmacology and Therapeutics : JPPT : the Official Journal of PPAG, 12(3), 138–13846. 10.5863/1551-6776-12.3.138 23055849 PMC3462096

[phy214999-bib-0008] Giraud, G. D., Morton, M. J., Reid, D. L., Reller, M. D., & Thornburg, K. L. (1995). Effects of ductus arteriosus occlusion on pulmonary artery pressure during in utero ventilation in fetal sheep. Experimental Physiology, 80(1), 129–139. 10.1113/expphysiol.1995.sp003828 7734132

[phy214999-bib-0009] Goolaub, D. S., Roy, C. W., Schrauben, E., Sussman, D., Marini, D., Seed, M., & Macgowan, C. K. (2018). Multidimensional fetal flow imaging with cardiovascular magnetic resonance: A feasibility study. Journal of Cardiovascular Magnetic Resonance, 20(1), 10.1186/s12968-018-0498-z PMC6264058 30486832

[phy214999-bib-0010] Groves, A. M., Kuschel, C. A., Knight, D. B., & Skinner, J. R. (2008). Does retrograde diastolic flow in the descending aorta signify impaired systemic perfusion in preterm infants? Pediatric Research, 63(1), 89–94. 10.1203/PDR.0b013e31815b4830 18043512

[phy214999-bib-0011] Grundy, D. (2015). Principles and standards for reporting animal experiments in the journal of physiology and experimental physiology. Journal of Physiology. 593(12):2547‐2549. 10.1113/JP270818 PMC4500341 26095019

[phy214999-bib-0012] Gulsun, M. A., Jolly, M. P., Guehring, J., Guetter, C., Littmann, A., Greiser, A., Markl, M., & Stalder, A. F. (2012). “A Novel 4D Flow Tool for Comprehensive Blood Flow Analysis.” In Proceedings of the 20th annual meeting International Society of Magnetic Resonance in Medicine. Melbourne, Australia, 1176pp.

[phy214999-bib-0013] Hooper, S. B., Te Pas, A. B., & Kitchen, M. J. (2016). Respiratory transition in the newborn: A three‐phase process. Archives of Disease in Childhood: Fetal and Neonatal Edition, 101(3), F266–F271. 10.1136/archdischild-2013-305704 26542877

[phy214999-bib-0014] Kilkenny, C., Browne, W., Cuthill, I. C., Emerson, M., & Altman, D. G. (2010). Animal research: Reporting in vivo experiments: The ARRIVE guidelines. British Journal of Pharmacology, 160(7), 1577–1579. 10.1111/j.1476-5381.2010.00872.x 20649561 PMC2936830

[phy214999-bib-0015] Kluckow, M., & Evans, N. (2000). Low superior vena cava flow and intraventricular haemorrhage in preterm infants. Archives of Disease in Childhood: Fetal and Neonatal Edition, 82(3), F188–F194. 10.1136/fn.82.3.f188 10794784 PMC1721081

[phy214999-bib-0016] Loecher, M., Schrauben, E., Johnson, K. M., & Wieben, O. (2016). Phase unwrapping in 4D MR flow with a 4D single‐step laplacian algorithm. Journal of Magnetic Resonance Imaging, 43(4), 833–842. 10.1002/jmri.25045 26417641

[phy214999-bib-0017] Luz, Z., & Meiboom, S. (1963). Nuclear magnetic resonance study of the protolysis of trimethylammonium ion in aqueous solution‐order of the reaction with respect to solvent. The Journal of Chemical Physics, 39(2), 366–370. 10.1063/1.1734254

[phy214999-bib-0018] Markl, M., Frydrychowicz, A., Kozerke, S., Hope, M., & Wieben, O. (2012). 4D flow MRI. Journal of Magnetic Resonance Imaging, 36(5), 1015–1036. 10.1002/jmri.23632 23090914

[phy214999-bib-0019] Roberts, T. A., van Amerom, J. F. P., Uus, A., Lloyd, D. F. A., van Poppel, M. P. M., Price, A. N., Tournier, J.‐D., Mohanadass, C. A., Jackson, L. H., Malik, S. J., Pushparajah, K., Rutherford, M. A., Razavi, R., Deprez, M., & Hajnal, J. V. (2020). Fetal whole heart blood flow imaging using 4D cine MRI. Nature Communications, 11(1), 1–13. 10.1038/s41467-020-18790-1 PMC7536221 33020487

[phy214999-bib-0020] Saini, B. S., Darby, J. R. T. Portnoy, S., Sun, L., Amerom, J., Lock, M. C., Soo, J. Y., Holman, S. L., Perumal, S. R., Kingdom, J. C., Sled, J. G., Macgowan, C. K., Morrison, J. L., & Seed, M.. 2020. Normal human and sheep fetal vessel oxygen saturations by T2 magnetic resonance imaging. The Journal of Physiology, 598(15), 3259–3281. 10.1113/JP279725 32372463

[phy214999-bib-0021] Sankar, M. N., Bhombal, S., & Benitz, W. E. (2019). PDA: To treat or not to treat. Congenital Heart Disease, 14(1), 46–51. 10.1111/chd.12708 30811796

[phy214999-bib-0022] Schrauben, E. M., Darby, J. R. T., Saini, B. S., Holman, S. L., Lock, M. C., Perumal, S. R., Seed, M., Morrison, J. L., & Macgowan, C. K. (2020). Technique for comprehensive fetal hepatic blood flow assessment in sheep using 4D flow MRI. The Journal of Physiology, 598(17), 3555–3567. 10.1113/JP279631 32533704

[phy214999-bib-0023] Schrauben, E. M., Lim, J. M., Goolaub, D. S., Marini, D., Seed, M., & Macgowan, C. K. (2019). Motion robust respiratory‐resolved 3D radial flow MRI and its application in neonatal congenital heart disease. Magnetic Resonance in Medicine, 83(2), 535–548. 10.1002/mrm.27945 31464030

[phy214999-bib-0024] Schrauben, E. M., Saini, B. S., Darby, J. R. T., Soo, J. Y., Lock, M. C., Stirrat, E., Stortz, G., Sled, J. G., Morrison, J. L., Seed, M., & Macgowan, C. K. (2019). Fetal hemodynamics and cardiac streaming assessed by 4D flow cardiovascular magnetic resonance in fetal sheep. Journal of Cardiovascular Magnetic Resonance, 21(1), 1–11. 10.1186/s12968-018-0512-5 30661506 PMC6340188

[phy214999-bib-0025] Shepherd, J. L., & Noori, S. (2019). What Is a Hemodynamically Significant PDA in Preterm Infants? Congenital Heart Disease, 14(1), 21–26. 10.1111/chd.12727. 30548469

[phy214999-bib-0026] Sung, S. I. (2019). Controversy in the diagnosis and treatment of hemodynamically significant patent ductus arteriosus in preterm infants. Korean Journal of Pediatrics, 62(11), 410–411. 10.3345/kjp.2019.00570 31319651 PMC6881201

[phy214999-bib-0027] Vonderen, J. J., Van, A. B., Pas, T. E., Kolster‐Bijdevaate, C., Van Lith, J. M., Blom, N. A., Hooper, S. B., & Roest, A. A. W. (2014). Non‐invasive measurements of ductus arteriosus flow directly after birth. Archives of Disease in Childhood: Fetal and Neonatal Edition, 99(5), 10.1136/archdischild-2014-306033 24966129

